# Gorham’s Disease of the Maxilla and Mandible With Distinctive Cone Beam Computerized Tomographic Features

**Published:** 2017-07-01

**Authors:** Rupam Sinha, Soumyabrata Sarkar, Tanya Khaitan, Deepsikha Ramani

**Affiliations:** 1 *Dept. of Oral Medicine & Radiology, Haldia Institute of Dental Sciences and Research, Haldia, West Bengal, India*; 2 *Dept. of Dentistry, Murshidabad Medical College and Hospital, Berhampore, West Bengal, India*

**Keywords:** Gorham’s disease, Maxilla, Mandible, Massive Osteolysis, Resorption

## Abstract

Gorham’s disease is a rare and atypical disorder epitomized by progressive osteolysis of bone with eventual total disappearance of bone. The etiology is poorly understood with variable clinical presentation. Most times it is initially misdiagnosed as temporomandibular joint dysfunction, periodontal disease or odontogenic tumors clinically and radiographically in routine dental practice. Radiographic examination, such as Cone Beam Computerized Tomography (CBCT) play a vital role in diagnosing such disorder resulting in disappearance of the involved bone entirely, which is a definitive distinguishing feature of this condition. Regarding the rarity of the condition, the current study presents a case of Gorham’s disease with distinctive clinical, radiological, and histological, features involving maxilla and mandible.

## Introduction

Gorham’s disease is an uncommon disorder of ambiguous etiology, characterized by spontaneous and progressive massive osteolytic event involving one or more bones. The disorder envisages incident of ultimate osteoporosis ensuing complete dissociation in normal coupling mechanism of the involved bone, resulting in rapid destruction of osteoid matrix followed by proliferation of angiogenic components. Despite its clinical aggressiveness it is mostly self-limiting and rarely fatal (1).

Gorham’s disease is among few clinical entities, which are described under numerous headings, due to its obfuscating and diversifying etiopathological presentation. These entities include Gorham-Stout Syndrome, Morbus Gorham-Stout Disease, Massive Osteolysis, Idiopathic Massive Osteolysis, Progressive Massive Osteolysis, Vanishing Bone Disease, and Phantom Bone Disease (2). The disorder came to light in medical literature in 1838 (Jackson), later on in 1924, a similar case occurring in jaws was reported by Romer. Gorham in 1954 and Gorham and Stout in 1955 presented a case series and portrayed this disorder as an unambiguous pathological entity (3).

International literature revealed that less than 200 cases have been reported with about 51 cases involving the oral and maxillofacial site to date (4). Radiographic findings are a key to diagnosis of this entity as first described by Johnson and McClure in 1958. Frederiksen et al. reported the mandible to be the most common site followed by maxilla with varying degrees of involvement of neighbouring structures, including the hard palate, sphenoid bone, zygomatic bone, etc. Heffeze *et al*. (1983) described the diagnostic criteria for massive osteolysis as follows: a) minimal or no osteoblastic response and absence of dystrophic calcification, b) positive biopsy for angiomatous tissue, c) absence of cellular atypia, d) evidence of local progressive osseous resorption, e) non-expansile, non-ulcerative lesion, f) absence of visceral involvement, g) osteolytic radiographic pattern, and h) negative hereditary, metabolic, neoplastic, immunologic or infectious etiology (4). The present study reports a rare case of Gorham’s disease in a 38-year-old male with distinctive clinical, radiological, and histological characteristics, involving maxilla and mandible.

## Case Report

A 38-year-old male referred with the complaint of partial edentulousness in the lower jaw and intended to get it replaced. The patient gave history of mobility of lower teeth, which gradually became exfoliated in the span of the last 10 years. He had previously consulted multiple dental surgeons for the same issue, where the remaining mobile teeth were extracted. The patient noticed that his lower jaw was becoming thinner progressively after extraction. There was negative history of pain, blood or purulent discharge from the associated region. Medical, family, and personal history were found to be inconspicuous.

**Figure 1:**
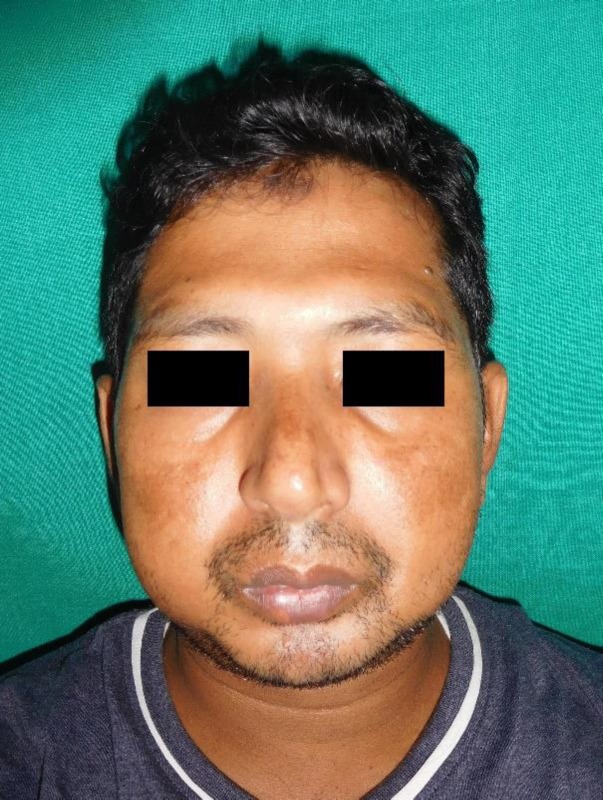
Clinical photograph showing decreased vertical height of lower one third of face.

**Figure 2:**
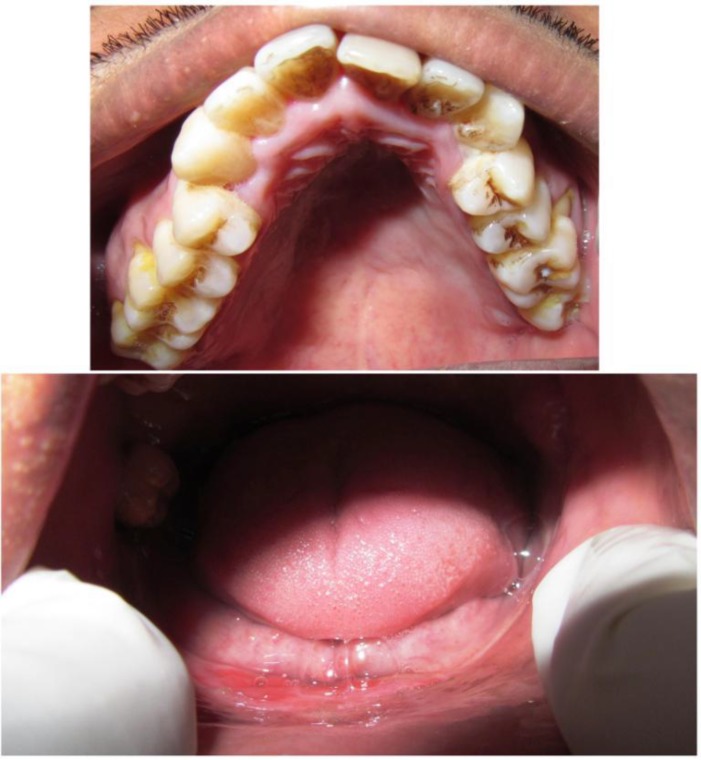
Intraoral photograph showing completely resorbed mandibular arch and periodontally compromised teeth in maxillary arch.

On extraoral examination, retrognathic profile, decreased vertical height of lower one-third of the face was noted (Figure 1). Intraoral examination revealed the presence of only third molars in completely resorbed mandibular arch. All the teeth were present in maxillary arch and were periodontally weak (Figure 2). Based on history and clinical features, a provisional diagnosis of osteolytic lesion of mandible was considered.

**Figure 3:**
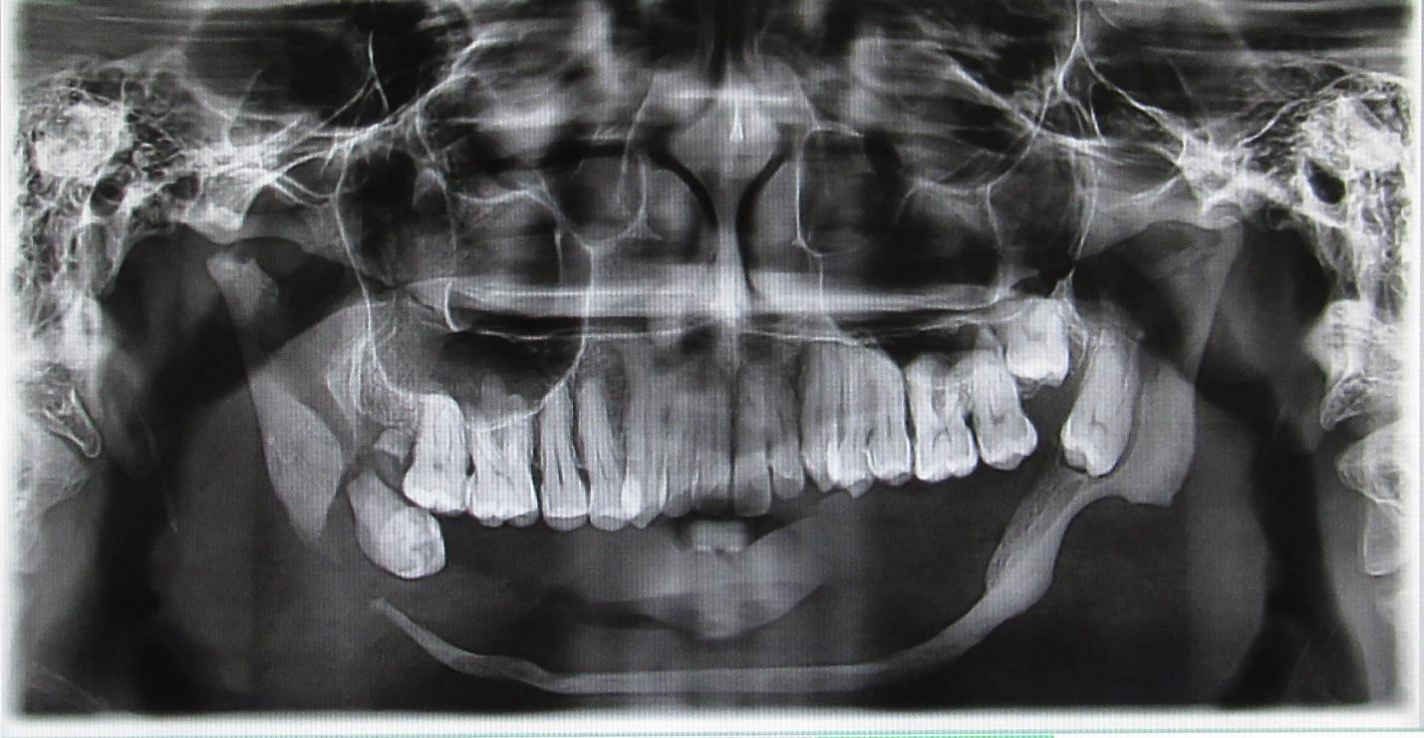
Orthopantomograph showing resorption of the mandibular arch and pathological fracture in the right lower border of mandible.

**Figure 4:**
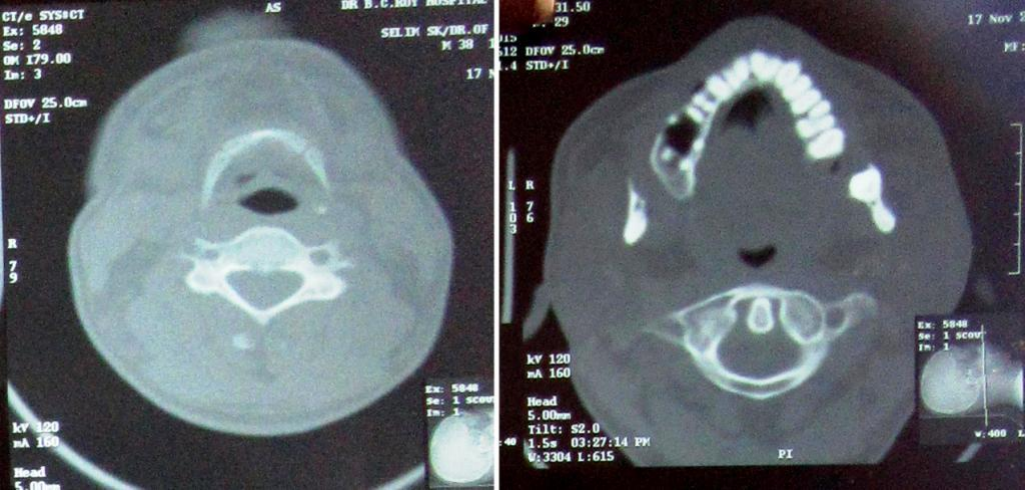
Axial section of computed tomographic image showing almost completely resorbed lower arch along with hypodense areas in right upper maxilla.

**Figure 5:**
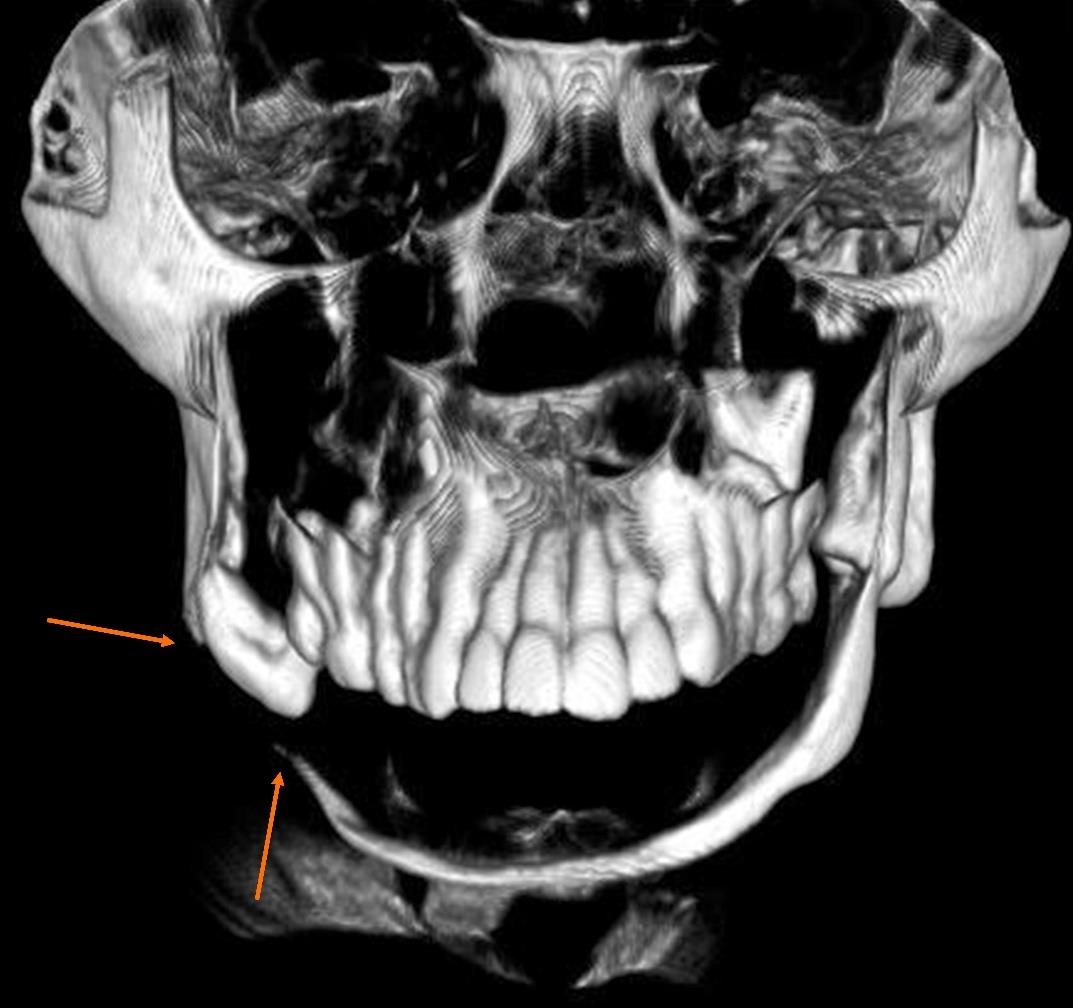
Coronal 3D-CBCT image showing resorbed mandible and pathological fracture of right lower border of mandible (orange arrows).

**Figure 6:**
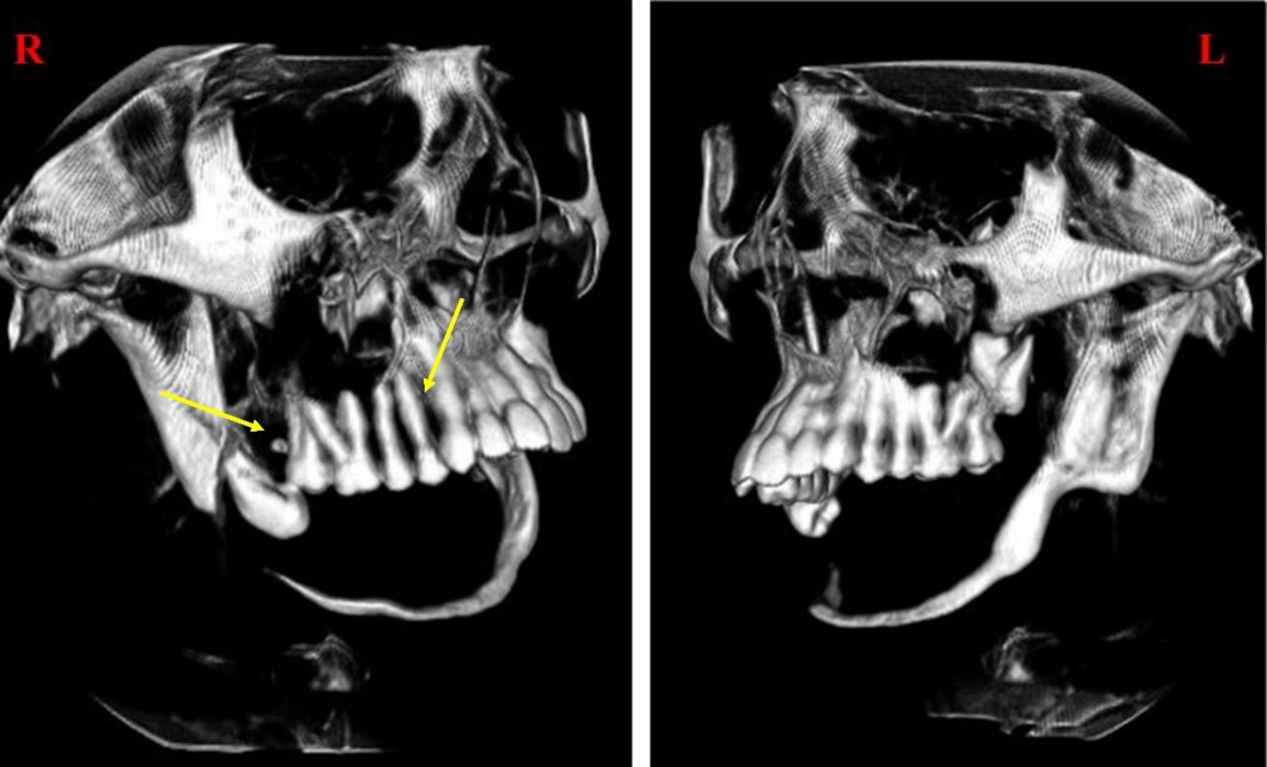
Sagittal 3D-CBCT image showing hypodense areas in right posterior maxillary region (yellow arrows) and completely resorbed mandible.

**Figure 7:**
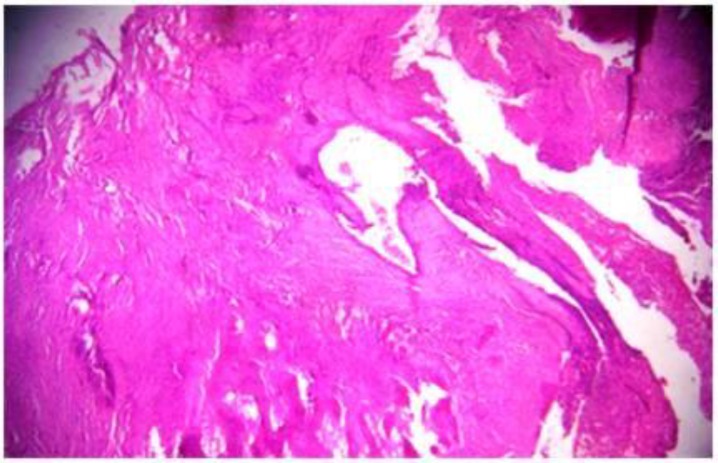
Photomicrograph (10X) showing thinned out bony matrix, vascular spaces and dense fibrous connective tissue stroma.

The patient was subjected to radiological, laboratory, and histopathological investigations. Orthopantomograph showed resorption of the mandibular arch with decreased vertical height of the mandibular body leaving a thin rim and discontinuation in the right lower border of mandible suggestive of pathological fracture (Figure 3). Axial section of computed tomographic image revealed almost completely resorbed lower arch along with hypodense areas in right upper maxilla (Figure 4). Coronal and sagittal sections of the cone beam computerized tomographic (3D-CBCT) image showed hypodense areas in right posterior maxillary region and thin rim of alveolar ridge, observed throughout the length of the mandible. There was loss of integrity of right lower border of mandible, indicating pathological fracture (Fig 5 and Fig 6). Laboratory investigations revealed normal blood picture except for elevated acid phosphatase and decreased alkaline phosphatase levels. Incisonal biopsy was performed from the left mandibular region and the photomicrograph revealed thinned out bony matrix, vascular spaces, and fibrous connective tissue stroma with dense collagenous bundles (Fig 7). Based on the history, clinical findings, and investigations, a final diagnosis of Gorham’s disease was confirmed.

The patient was explicated about the rapidly progressive nature of the disease and recieved counselling and support as there is currently no definitive treatment.

## Discussion

Gorham disease is an atypical pathological entity presented by spontaneous and usually progressive destruction of one or more bones. The destroyed bone is replaced by vascular proliferation, which is eventually interchanged by dense fibrous tissue. The most commonly affected bones are clavicle, scapula, humerus, ribs, and sacrum. Involvement of maxillofacial bones is very rare (2,3).

The exact cause and nature of the disease process still remains irresolute. The normal bone is replaced by expanding non-neoplastic vascular tissue. The presence of slow flowing blood through the wide capillary-like vessels produces local hypoxia and reduced pH thus favouring the activity of various hydrolytic enzymes, like acid phosphatase and leucine aminopeptidase. These enzymes, which are in contact with bone are suggestive of the osseous resorptive property of these cells. Gorham and Stout hypothesized that vascular granulation tissue production may be stimulated due to trauma and stated that “osteoclastosis” is not necessary.(4)

Dickson et al. (1987) and Devlin et al. (1996) recommended that osteoclastic activity is enhanced by mononuclear phagocytes, multinuclear osteoclasts, and vascular endothelium, and triggers interleukin 6 that helps in bone resorption in this disorder. In 1998, Korsic et al. elucidated that deficiency of C cells and subsequent lack of calcitonin could result in this disorder. Hirayama et al. (2001) and Colucci et al. (2006) postulated that the increase in osteoclast is due to an increase in the sensitivity of the osteoclast precursors (from monocyte-macrophage lineage) to humoral factors, which promote osteoclast formation and bone resorption owing to its osteoclastogenic and angiogenic molecules. Hagendoorn et al. (2006) signified the critical role of the PDGFR-b, which initiates the signalling pathway (receptor of the lymphangiogenic growth factor, Platelet Derived Growth Factor BB), contributing to pathogenesis (4,5).

Gorhams disease affects all ages, ranging from 1 month to 66 years with 2:1 male to female ratio. The average presenting age is 29.6 years with facial bone involvement. No family history of any bone pathology or developmental abnormality is observed. Mandible is more commonly involved as compared to maxilla; individual maxillary bone involvement has not been reported (6). In the above case, involvement of mandible followed by maxilla was observed.

Clinically, two sequential phases have been detected in this disease. In the initial phase, there is active bone destruction and lysis coupled with mild to moderate pain, soft tissue distension, tenderness, and varying degrees of erythema followed by radiographic evidence of progressive bone destruction. The second phase is characterised by absence of swelling, pain, no difficulty in performing daily activities, bone destruction or radiographic evidence of bone repair (6). Spontaneous fractures may occur and lack of bone healing following fracture is the hallmark of the disease (7). In the current case, the findings were consistent with the second phase.

 Radiographically, radiolucent foci appear in the initial phase in intramedullary or subcortical regions with undisturbed margins, which unite eventually involving the cortex followed by slow progressive resorption, dissolution, fracture, disintegration, and desertion of a portion of the involved bone. Progressive involvement reveals ‘‘tapering’’ or ‘‘pointing’’ of the residual osseous tissue and deterioration of soft tissues (8). Patrick in 1976 classified the radiological changes in 4 stages: a) radiolucent foci resembling patchy osteoporosis, b) shrinkage of shaft of bones by tapering of ends (sucked candy appearance), c) complete resorption of bone unless there is spontaneous arrest, and d) progression to adjacent joints and bones (9). The present case findings were in concordance with the fourth stage as mandible involvement was followed by maxilla.

Advanced imaging modalities, such as computed tomography and magnetic resonance imaging played a chief role in evaluation of the involvement of facial bones and the skull, and determination of any extension into the base of the skull. Radioisotopic scan demonstrates initial increased vascularity followed by an area of decreased uptake equivalent to the site of diminished or deficient osseous tissues (3). The CBCT has emerged as a novel advanced imaging technology in diagnosing bony pathologies with outstanding results. In the present case, 3D-CBCT had been performed, which revealed the exact nature and involvement of maxilla and mandible.

Although the extent of osseous deformity might become severe, serious complications are uncommon. Thoracic cage, pulmonary or pleural involvement can result in compromised airway and death (4,5,7).

There is no definitive therapy available for Gorham’s disease. Radiation therapy, anti-osteoclastic medication (bisphosphonates), alpha-2b interferon, adrenal extracts, androgens, and surgery with reconstruction using bone grafts have been attempted. Definitive radiotherapy in moderate doses (40 to 45 Gy in 2 Gy fractions) resulted in constrained osteolytic activity with few enduring complications (10).

## Conclusion

Gorham’s disease is an atypical musculoskeletal disarray with speculative etiology and erratic clinical presentation. Most of the times, it is misdiagnosed initially as temporomandibular joint dysfunction, periodontal disease or odontogenic tumors, clinically and radiographically (3,11). Therefore, to conclude, the current case presents with the clinicopathological events of Gorham’s disease putting emphasis on CBCT features as well as on the role of dental practitioner, as a dental surgeon might be the first healthcare provider for patients seeking medical care for such disorders.
